# Ethical and procedural issues for applying researcher-driven multi-national paediatric clinical trials in and outside the European Union: the challenging experience of the DEEP project

**DOI:** 10.1186/s12910-021-00618-2

**Published:** 2021-04-29

**Authors:** Viviana Giannuzzi, Mariagrazia Felisi, Donato Bonifazi, Hugo Devlieger, George Papanikolaou, Lamis Ragab, Slaheddine Fattoum, Bianca Tempesta, Giorgio Reggiardo, Adriana Ceci

**Affiliations:** 1 Fondazione per la Ricerca Farmacologica Gianni Benzi Onlus, Via Abate Eustasio, 30, 70010 Valenzano, Italy; 2 Consorzio Per Valutazioni Biologiche E Farmacologiche (CVBF), Via Luigi Porta, 14, 27100 Pavia, Italy; 3 Consorzio Per Valutazioni Biologiche E Farmacologiche (CVBF), Via Nicolò Putignani 178, 70122 Bari, Italy; 4 Department of Development and Regeneration, Faculty of Medicine, Catholic University of Leuven (KULeuven), Herestraat 49, 3000 Leuven, Belgium; 5 Pathophysiology and Human Genetics, Harokopion University, 70 El. Venizelou, 17671 Athens, Greece; 6 Life and Health Sciences, Cairo University, Al Orman Guiza, Giza, 12613 Egypt; 7 Faculty of Medicine, University of Tunis El Manar, Campus Universitaire Farhat Hached, B.P. n° 94 - ROMMANA, 1068 Tunis,, Tunisia; 8 Data Manager and Biostatistician, Medi Service, Via De Marini, 1, 16149 Genova, Italy

**Keywords:** Clinical trial application; Multi-national trial; Paediatric clinical research; Biomedical ethics

## Abstract

**Background:**

We describe our experience from a multi-national application of a European Union-funded research-driven paediatric trial (DEEP-2, EudraCT 2012-000353-31; NCT01825512). This paper aims to evaluate the impact of the local and national rules on the trial authorisation process in European and non-European countries. National/local provisions and procedures, number of Ethics Committees and Competent Authorities to be addressed, documentation required, special provisions for the paediatric population, timelines for completing the authorisation process and queries received were collected; compliance with the European provisions were evaluated. Descriptive analysis, Wilcoxon Rank-Sum test and General Linear Model analysis were used to determine factors potentially influencing the timelines. The Cluster Analysis procedure was used to identify homogenous groups of cases.

**Result:**

The authorisation process was completed in 7.7 to 53.8 months in European countries and in 17.1 to 27.1 months in non-European countries. The main factors influencing these timelines were the requests for changes/clarifications in European countries and the different national legislations in non-European countries.

**Conclusion:**

This work confirms that the procedures and requirements for the clinical trial application of a paediatric trial are different. In the European Union, the timeframes for submission were generally harmonised but longer. In non-European countries, delays were caused by national dispositions but the entire authorisation process resulted faster with less requests from ECs/CAs. The upcoming application of Regulation (EU) 536/2014 is expected to harmonise practices in Europe and possibly outside. Networks on paediatric research acting at international level will be crucial in this effort.

**Supplementary Information:**

The online version contains supplementary material available at 10.1186/s12910-021-00618-2.

## Background

Trials involving ‘small populations’, such as children and patients affected by rare diseases, are typically multi-centre and multi-national. For this type of studies, the preparation of the Clinical Trial Application (CTA) can be a difficult enterprise [1]. In fact, besides Good Clinical Practice (GCP) guidelines [2], the different rules and procedures set out at a local level need to be complied with. The available guidance does not address the specific provisions for carrying out a multi-national trial [1].

In Europe, GCP has been implemented by two different Directives [3, 4]. Directive 2001/20/EC [3] introduced the obligation to obtain a Competent Authority (CA) “authorisation” and offered the opportunity to mandate a single opinion from the coordinating centre in case of multi-national trials. In this case, the other sites, as ‘satellite’ sites, were required only to grant the acceptance/refusal of the single opinion.

Furthermore, the European Commission released two documents, specifically issued to guide the submission of clinical trials to Ethics Committees (ECs) [5] and CAs [6] and to prepare documents for a multi-national CTA. Directive 20/2001/EC [3] and related guidance, were aimed to harmonise and, when possible, to reduce the burden of the procedures. However, their legislative power is limited [1].

The provisions specifically addressed to paediatrics coming from directives, guidelines and recommendations have been implemented even more differently at national level [7]. Among them, the Ethical Recommendations [8] as updated in 2017 [9] provides recommendations on benefit/risk assessment, protocol designs and populations, the ethical review of paediatric protocols from experts, insurance, the involvement in the informed consent/assent process of children and their rights [10] and they are intended to be applied also outside the EU. They recommend to inform paediatric trial participants according to their age and maturity and to collect their informed assent. This concept has been adopted by the new CT Regulation [13] and recently entered into force in the EU.

The need for harmonising CTA procedures for multi-centre trials has also been emphasised outside the European Union (EU). As an example, Matheson and colleagues claimed for a more effective and efficient approach to multicentre paediatric research because the current disparate system places too large a burden on both the investigators involved in multicentre studies and the local ECs and therefore proposed a common ethics application form and consenting process to harmonise the ethics review process for multicentre paediatric studies in Canada [11]. In Russia, changes in legislation were made to make the process of clinical trial approval better defined and more transparent, thus, contributing to a decrease in the administrative burden [12].

In other non-EU countries such as Albania, Egypt and Tunisia, the context is different: generally, the ICH-GCP have been implemented but clinical trials legislation and/or a specific paediatric medicines regulation is lacking.

Furthermore, it should be noted that the ethical issues and procedures in one site of the country do not necessarily represent the situation of the country as a whole, as each ethics committee can have its own rules and follow different procedures asking different documents and contents of the CTA.

This results in a greater effort to guarantee an ethical standard among trial sites in the case of a multi-national paediatric study involving EU and non-EU countries. For instance, the written and oral information should be guaranteed in the same way in all centres and customised according to the cultural, political and social settings.

Another key and largely discussed issue related to CTA is the long timeline needed to obtain the full authorisation, often far from the provision set out by Directive 2001/20/CE, i.e., 60 days for the CA authorisation and single opinion release and 30 days for its acceptance [3]. In fact, criticisms on these issues have led to the release of a EU Clinical Trials Regulation [13].

We performed a multi-centre, randomised, open-label, non-inferiority active-controlled efficacy-safety trial involving paediatric patients from 1 month to less than 18 years affected by transfusion-dependent haemoglobinopathies (DEEP-2; EudraCT 2012–000353-31; NCT01825512) according to a Paediatric Investigation Plan (PIP) agreed by PDCO (P/0357/2016). The study is part of the EU funded project “DEferiprone Evaluation in Paediatrics” (DEEP, FP7 HEALTH-F4-2010-261483) [14, 15]. The study was based on the setting up of a unique submission package to ECs and CAs based on GCP and other specific paediatric EU requirements [8], case by case adaptation of this package according to the national frameworks in force at the time of the submission in each involved country.

The aim of this paper is to evaluate the impact of the different local and national rules and procedures and of their complexity on the paediatric trial authorisation process in different EU and non-EU countries in terms of timelines for the final EC approval and CA authorisation from the preparation of submission package to the release of authorisation and approval.

## Methods

### What

DEEP-2 CTA procedure was performed in 23 trial sites, of which 17 were located in the EU (Cyprus, Greece, Italy and the UK) and 6 in non-EU countries (Egypt, Albania and Tunisia) that include 7 coordinating sites (located in the 7 concerned countries) and 15 satellite sites.

### How

To meet the aim of the study, we collected the following information for each site:

1. Applicable national legal frameworks ruling the CTA procedures, timelines and required documents.;
2. Local procedures to authorise a paediatric trial in force at the time of the CTA. The following parameters were considered:
  - The number of ECs and CAs to be addressed;
  - If the submission is to be carried out in parallel or subsequently to the CA and the EC;
  - Preparation of the CTA form;
  - Number and type of documentation required for the CTA.

Compliance of required documents with the EU provisions was assessed by comparing the lists of documents required for the submission in the seven participating countries with the documents required by the EC guidance [5, 6].

The complexity of the procedure was based on the number of required submissions, type of submission (if performed subsequently to the CA and the EC), preparation of the CTA form (if done through or outside the EU platform, as detailed below), and the number of documents required by EC/CAs which exceed the EC requirements.

We also analysed the number and the contents of queries received from the CAs and ECs.

Finally, the performance of the whole process was evaluated in terms of timelines referring to the following slots:

- Time from the submission package ready to package submitted to CAs and/or ECs;
- Time from submission to CAs/ECs authorisation/opinion granted.

In particular, the first slot included the timeframe spent from the sponsor for the administrative/legislative procedure until the submission package is formally submitted. This period was necessary to adapt the “master” submission package to all the local requirements including the preparation of site-specific documents and all the necessary local authorisations propaedeutic to the submission in addition to the EC approval and CA authorisation.

We considered the timelines related to the coordinating trial sites separately from the satellite sites.

For the analysis, we grouped 7 countries and 23 trial sites into two groups: EU and non-EU, considering that the countries of the EU have a common legal framework that would make rules more harmonised.

### Sources of information

Local procedures to authorise a paediatric trial in the seven countries involved were primarily derived by a survey addressed to DEEP-2 local study teams (Additional file 1: Table S1). Any possible further detail on laws, requirements for minors, provisions for data protection and confidentiality and requested documents were retrieved from official sources of ECs and CAs [15] and from the Inventory of procedures for obtaining clinical trial authorisations from TEDDY [16].

The submission dates, number and type of local/national documentation submitted in each centre, as well as the requests for modifications/clarifications from CAs and ECs were provided by DEEP-2 sponsor as sourced by the Trial Master File and other study documentation.

### Statistical analysis

The Wilcoxon Rank-Sum test, a non-parametric analogue of the two samples t-test based on ranks of data, was used to compare median between two independent samples (EU countries versus non-EU countries) without the assumption of normally distributed data.

The General Linear Model (GLM) analysis was used to identify possible predictors that could explain the differences in ECs approval time in the group of DEEP-2 study centres. Thus, GLM analysis provided regression analysis and analysis of variance for one dependent variable (ECs approval time) by two factors (EU or non-EU countries, national coordinating centre or satellite sites) and one covariate (number of requests by ECs).

The Cluster Analysis was used to identify groups of CTA sites behaving similarly with respect to the timeframe of authorisation. This procedure was used to check in an independent way the hypothesis that EU and non-EU CTA sites have significantly different timeframes of authorisation, without knowing a priori where the sites are located.

### When

The CTA was performed in the period June 2012 to September 2015 in 23 trial sites. The EC approvals and CA authorisations were released in the period January 2013 to February 2015.

## Results

The questionnaire on local procedures to authorise a paediatric trial was sent to seven DEEP-2 local study teams. Feedback from seven was received, thus covering all the seven countries involved in the trial recruitment.

### National legal framework

As shown in Table 1, in all EU countries participating in the DEEP-2 study, national laws ruling the CTA have implemented EU Directives [3, 4] and GCP were applied.

**Table 1:** National rules on the CTA in the DEEP-2 countries

Country	National rules	National rule on paediatric trial	GCP application
*EU*			
Cyprus	YES according to Directive 2001/20/EC [3]	YES	Implemented in the national Law
Greece	YES according to Directive 2001/20/EC [3]	YES	Implemented in the national Law
Italy	YES according to Directive 2001/20/EC [3]	YES	Implemented in the national Law
UK	YES according to Directive 2001/20/EC [3]. Besides the EC approval and CA authorisation, another approval from the CRN-NHS highly recommended	YES	Implemented in the national Law
*Non-EU*			
Albania	NO	NO	Not implemented
Egypt	YES but national law prevents to move biological samples beyond the borders	YES	Implemented but no legislative disposition exists
Tunisia	YES	paediatric trials with a non-marketed IMP not allowed	Implemented but no legislative disposition exists

In non-EU countries, ad hoc national rules were in place at the time of DEEP-2 submission with the exception of Albania. GCP were not ‘officially’ implemented in a national law in Egypt and Tunisia, but currently implemented in the trials conducted. In Albania, GCP principles were accepted and implemented for the first time in that country as a prerequisite to participating in the DEEP-2 study.

In all the EC countries, paediatric trials are accepted and included in the scope of directives and national laws. With reference to non-EU countries, in Egypt paediatric trials were allowed while in Tunisia, under normal circumstances, paediatric trials with a non-marketed IMP were not allowed, and special authorisation from the Tunisian Ministry of Health to perform DEEP-2 trial was required and finally granted after written proves that this was part of a PIP agreed by the PDCO-EMA and sponsored by a not-for-profit consortium.

Special rules should be mentioned:

- In the UK, besides the EC approval and MHRA authorisation, another approval from the Clinical Research Network of the National Institute for Health Research (NHS) is highly recommended (non-mandatory);
- In Egypt, the national law prevents to move biological samples beyond the borders, unless a special authorisation is granted from the National Security.

Details of the national laws applicable in the DEEP-2 study countries are available at the DEEP project website [15].

### National/local requirements for DEEP-2 study authorisation process

Notably, the procedures of each site derived from the national rules, but each ethics committee and competent authority had their own procedures.

#### The number of ECs and CAs addressed per country

In three out of four EU countries (Cyprus, Greece and the UK), one EC and one CA were to be addressed, even in the case of multi-site applications. In Italy, one EC and one CA was addressed for each site for a total of 12 CAs and 12 ECs; the EC competent for the coordinating site granted the single opinion, and the other ECs granted the acceptance of the single opinion.

With regards to non-EU countries, in all of them (Albania, Egypt and Tunisia) a single CA authorisation was needed for all the sites involved at a national level. With reference to the ECs, for both Albanian sites, the EC approval was granted in two different period of time, due to the later inclusion of the second trial site. Therefore, for each site, a specific ethics approval was needed from two different ECs. In Egypt 1 EC application is needed in case of multi-site trials, while in Tunisia each site must have its ethics approval.

Further details are included in Additional file 1: Table S2.

#### Parallel or subsequent EC and CA submission

In three out of four EU countries (Cyprus, Greece and the UK), the EC and CA were addressed in parallel, while in Italy the CA authorisation was granted after the EC approval. With regards non-EU countries, in Albania, the application was carried out in parallel, while in Egypt and Tunisia, the CA authorisation was granted after the ethical approval. Further details are included in Additional file 1: Table S2.

#### Preparation of the CTA form

The document was prepared through the EU platform (https://eudract.ema.europa.eu/) for Cyprus and Greece, and through the national web-based platform for Italy and UK, i.e. the “*Osservatorio Nazionale per le Sperimentazioni Cliniche*” (OsSC) (10/12 submissions were web-based, 2/12 paper submissions because OsSC was temporarily closed due to technical issues) and the “*Integrated Research Approval System*” (IRAS) respectively.

In non-EU countries, a national CTA form was required; the EU CTA form for third countries was submitted as well, as required for trials included in a PIP [6]. Further details are included in Additional file 1: Table S2.

#### Documents required for the submission

EC guidance [5, 6] was considered as a reference to prepare the list of documents required at each site level. As detailed in Table 2, no substantial differences resulted between EU and non-EU countries. Notably, in no country was the assent form and information material for paediatric participants required by law despite the provisions contained in the EC Ethical Recommendations [8]. However, the assent form was mentioned in the list of documents for ECs in Italy, the UK and Egypt.

**Table 2:** Documents required for DEEP-2 submission compared to the EC Guidance [5, 6]^1^

Documents required by the EC Guidance		EU			Non-EU		
	CY	GR	IT^2^	UK	AL	EG	TU
*For EC approval*							
Cover letter, protocol, CTA form, information on countries and sites involved, synopsis in the national language, I.B./SmPC, insurance	**x**	**x**	**x**	**x**	**x**	**x**	**x**
Receipt of confirmation of EudraCT number, agreement between sponsor and site	**x**	**x**	**x**	**x**			
List of involved Competent Authorities	**x**	**x**	**x**^**5**^	**x**			
PIP opinion/link to the specific documentation^4^		**x**	**x**^**5**^	**x**			
IMPD, NIMPD (including GMP compliance)		**x**	**x**^**5**^	**x**	**x**	**x**	**x**
Examples of the label in the national language		**x**	**x**^**5;**6^	**x**	**x**	**x**	**x**
Outline of all active trials with the same IMP			**x**^**3**^				
Information documents for parents/legal representative, consent form, any other material used for the recruitment	**x**	**x**	**x**	**x**	**x**	**x**	**x**
Information material for children, assent form			**x**	**x**		**x**	
CVs of Investigators, Investigator disclosure of conflict of interest	**x**	**x**	**x**	**x**	**x**	**x**	
Quality of facilities for the trial	**x**	**x**	**x**			**x**	
*For CA authorisation*							
Cover letter, protocol, CTA form, contents of the labelling, I.B. /SmPC	**x**	**x**	**x**^**3**^	**x**	**x**	**x**	**x**
IMPD, NIMPD (including GMP compliance)	**x**	**x**	**x**	**x**	**x**		**x**
Insurance	**x**	**x**	**x**		**x**	**x**	**x**
EC opinion (where available)		**x**	**x**	**x**		**x**	**x**
PIP opinion/link		**x**	**x**	**x**			
Proof of payment	**x**	**x**	*n.a*	**x**		**x**	
Agreement between sponsor and site			**x**				**x**

n.a.: not applicable for DEEP-2 trial given the non-profit nature of the trial; IMPD: Investigational Medicinal Product Dossier; NIMPD: Not-Investigational Medicinal Product Dossier

^1^Scientific Advice, Indemnity/compensation for participants and rewards to investigators, unjustified/unexpected impurities, viral safety information, GMOs, radiopharmaceuticals, TSE certificate required by EC Guidance on ECs submission [5] not foreseen in DEEP-2 trial. Scientific Advice, Indemnity/compensation for participants and rewards to investigators required by EC Guidance on CAs submission [6] not foreseen in DEEP-2 trial

^2^12 CAs addressed in Italy

^3, 6^2 out of 12 sites in Italy

^4^ The PIP not mentioned in the Guidance [5], being issued after the Paediatric Regulation [17], but required by the EC

^5^ Only the EC issuing the single opinion

**Fig. 1:**
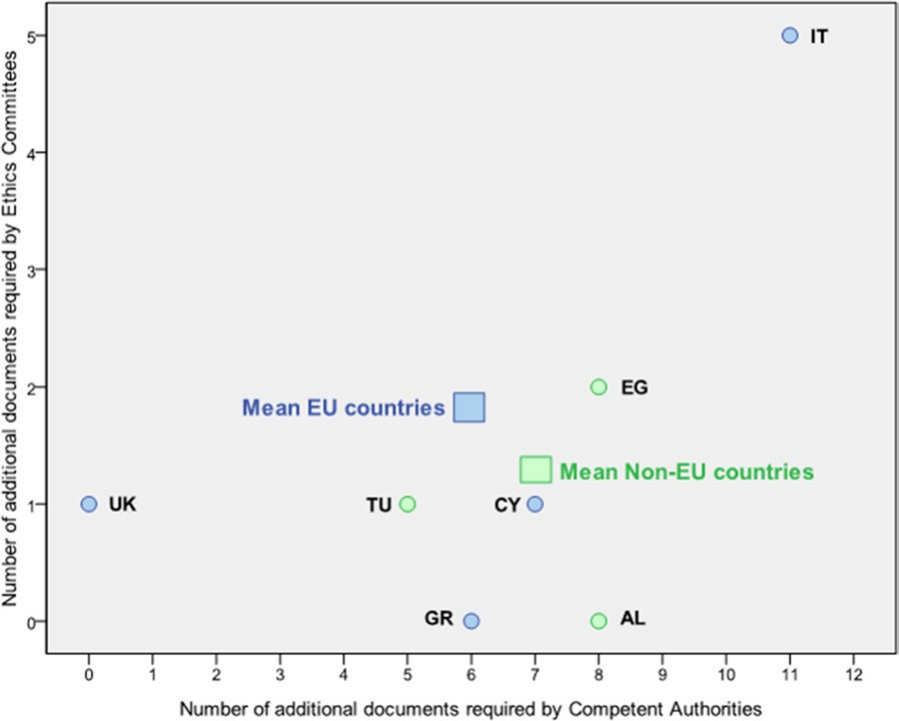
Number of additional documents required by Competent Authorities and Ethics Committees. This scatter-plot shows the number of documents required by each country in addition to those required by the EC guidance [5, 6] for each country and the means of EU and non-EU countries. For Italy, the numbers vary according to the 12 sites. For Egypt, the 3 ECs were considered. The numbers behind the country name are the site numbers

As shown in Fig. 1, in Italy the number of documents needed for submission was higher than the number of documents listed in the EU guidance.

### Queries during the authorisation process

Some CAs and ECs asked for clarifications and/or modifications to study documents, as detailed in Table 3. These were mainly requests for clarification. The request for changes were mostly related to national or local requirements.

**Table 3:** Requests raised by competent authorities and ethics committees

Request type	N. requests from ECs		N. requests from CAs	
	Tot	Details	Tot	Details
Changes/clarifications to/on material for patients/parents	87	Cyprus (1)	n.a	n.a
		Greece (1)		
		Italy (4)		
		UK (1)		
Changes/clarifications to/on protocol	3	Cyprus (1)	1	UK (1)
		Italy (1)		
		UK (1)		
Changes to/clarifications on insurance	3	Cyprus (1)	n.a	n.a
		Greece (1)		
		Italy (1)		
Other changes/clarifications^a^	4	Cyprus (1)	1	Egypt (1)
		Italy (3)		

The table shows the number of requests from 8 Ethics Committees and from 2 Competent Authorities as classified by type and country

n.a. not applicable: no request on that specific issue raised

^a^Study procedures, treatment after the study; non-profit declaration; financial contribution; sample exportation

The requests from the CAs were raised only in Egypt and the UK. With regards to the ECs, 8 out of 20 ECs, all from the EU, requested 18 changes/clarifications, mostly concerning the material for parents/patients. Table 4 shows the requests for changes on material for the informed consent and assent process by the ECs.

**Table 4:** Requests for changes on material for the informed consent and assent process from EU Ethics Committees

Request type	CY	GR	IT	UK
Inclusion of more details about Sponsor			**x**	
Inclusion of details on contraceptive methods			**x**	**x**
Inclusion of details on volume of blood sample			**x**	
Inclusion of information on anaesthesia and MRI	**x**		**x**	
Inclusion of information in risk and on insurance policy	**x**		**x**	
Inclusion of privacy of data (data will be remained confidential)	**x**			
Inclusion of information on the custody holder in the consent form	**x**			
Inclusion of detail on the taste of the new formulation				**x**
Inclusion of more information on pregnancy (test and management possible occurrence)				**x**
Removal of “thump prints” of participants and guardians from consent and assent forms		**x**		**x**
Replacing the booklets prepared by the sponsor with information sheets, rewording the information for 6–10 years old age group considered too complicated and splitting the Young Person Information Sheet for the 11 to 17 into two				**x**

Importantly, none of the concerned ECs raised issues on the protection of paediatric patients involved in the trial and none were related to ethical guidelines for the protection of research participants, including children.

However, in Cyprus, the trial was approved for not the entire paediatric population proposed in the protocol, i.e., from 1 month to less than 18 years, but from 2 years above.

### Timing for completing the authorisation procedures

**Fig. 2:**
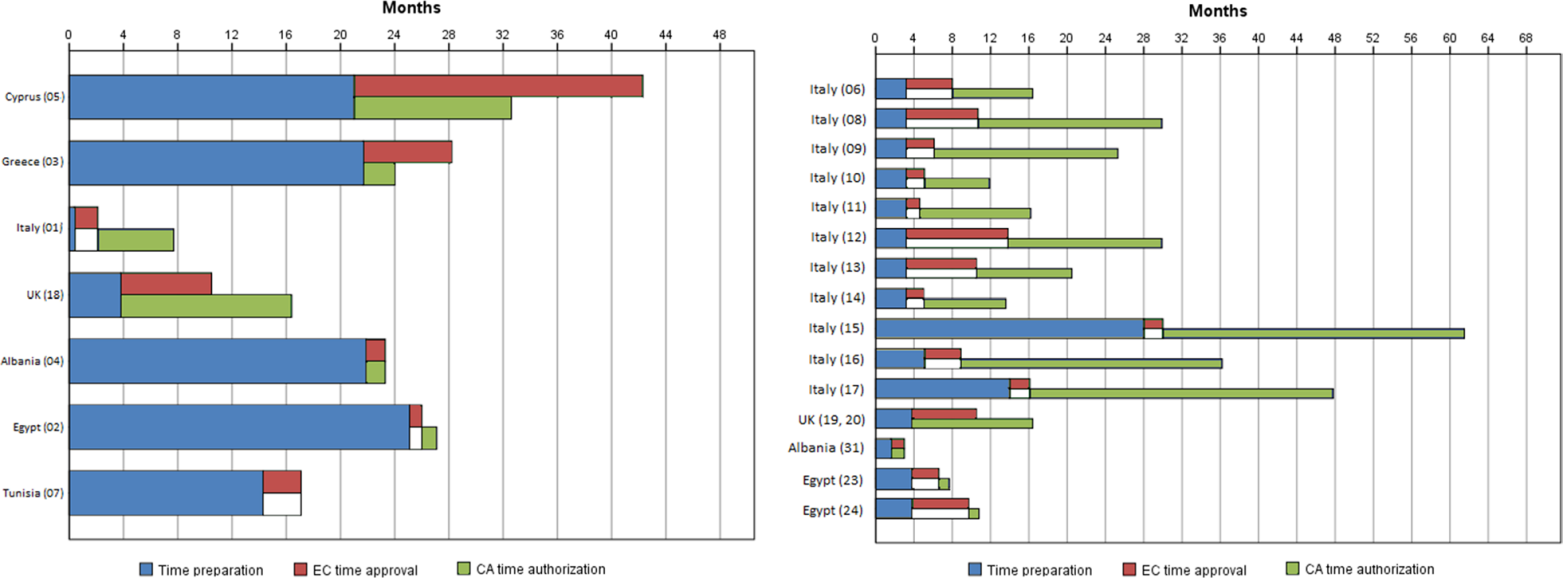
Time to obtain authorisation/approval by site. This figure shows the timing (months) for getting the CA authorisation and EC approval. The blue bars indicate the time necessary for preparing the CTA; the red and green bars indicate the time from the submission to the EC approval (red bars) and the CA authorisation (green bars). On the left side, data from the 7 countries is provided; on the right side, data is also provided for multi-site national submissions

Figure 2 describes the timing for getting the CA authorisation and EC approval divided in the two slots mentioned in the methods.

1. Time from the submission package ready to package submitted to CAs and/or ECs.
  With regards to the seven coordinating centres, the timing to prepare the submission package was ranging from 0.4 to 21.7 months (median = 11.7 months) in EU countries. In non-EU countries, this process was completed in a period ranging from 14.3 to 25.1 months (median = 20.4 months). With regards to the satellite sites, the preparation of the submission package was completed in 3.2–28 months (median = 6.3 months) in EU countries and from 1.6 to 3.8 months (median = 3 months) in non-EU countries.
2. Time from the submission to CA authorisation EC approval. With regards to the seven coordinating centres, the EC opinion and the CA authorisation were obtained in a period ranging from 7.3 to 32.9 months (median = 17.1 months) in the EU, and by 2.8 months (median = 2.5 months) in non-EU countries. With regards to the other sites, the EC opinion and the CA authorisation were obtained in a period ranging from 8.6 to 33.8 months (median = 21.3 months) in EU countries and from 2.8 to 7 months (median = 4.5 months) in non-EU countries.
  If we consider the entire process, the following data result. With regards to the seven coordinating centres, the timing ranged from 7.7 to 53.8 months (median = 28.8 months) in EU countries and from 17.1 to 27.1 months (median = 23 months) in non-EU countries. With regards to the other sites, this timing was ranging from 13.6 to 61.5 months (median = 27.7 months) in EU countries and from 4.4 to 10.7 months (median = 7.6 months) in non-EU countries.

### Statistical evaluation and cluster analysis

The GLM analysis (Additional file 1: Table S3) suggested that the ECs approval time was significantly correlated to the number of EC requests (*p* value < 0.001). When the statistical model is adjusted for the number of EC requests as covariate the difference observed in the comparison of the ECs approval time between EU and non-EU countries and between EC coordinating centre versus satellite sites was not significant, *p* value = 0.393 and 0.320 respectively.

It was possible to hypothesise that the difference in the ECs approval time observed between EU and non-EU countries is related to the greater number of the requests of the EU ECs. The cluster analysis identified two clusters (EU and non-EU) (Additional file 1: Figure S1). Longer authorisation period resulted in the EU and a shorter authorisation period for non-EU. The comparison of the CA time authorisation in months showed a significant difference between EU and non-EU clusters (Wilcoxon Rank-Sum test *p* value = 0.001; Additional file 1: Figure S1). In the same way, longer EC time approval resulted for coordinating centres (mean = 5.9 months) and shorter time approval period for satellite sites (mean = 4.2 months), Wilcoxon Rank-Sum test *p* value = 0.699. The CA time authorisation showed an opposite trend comparing the ECs: longer CA time authorisation resulted for satellite sites (mean = 14.8 months) and shorter time authorisation for coordinating centres (mean = 4.9 months), Wilcoxon Rank-Sum test *p* value = 0.046.

## Discussion

The preparation and submission to ECs and CAs, as established by art. 9 of Directive 2001/20/EC [3] for multi-national studies is a challenging duty for the sponsor. In fact, each country has its own rules and procedures to comply with, and unfortunately, even each EC can have their own, while an ethical standard among trial centres should be guaranteed at the same time. In this work, we described our experience of a multi-centre multi-national CTA in EU and non-EU countries with different cultures, languages, geographical and political frameworks, as well as different pharmaceutical systems and prescriptive habits [18]. In fact, notwithstanding the implementation of EU Directives into national laws and the acknowledgment of the EU system and guidance, differences in regulatory frameworks and procedures when applying a multi-national trial still remain.

On the other hand, the framework of non-EU countries for paediatric clinical trials is not yet implemented, thus leading to legal obstacles during the preparedness of a trial. Noticeably, DEEP-2 has represented the first paediatric clinical trial carried out in Albania, where Law No. 9323/2004 on Drugs and Pharmaceutical Service [19] which defines the rules of pharmaceutical service and medicines production, trading, prescribing, quality control, and inspection, was not specifically addressed to clinical trials.

In fact, the preparation of the CTA for coordinating sites, as the first to be addressed, was longer in non-EU countries. For EU countries, the shortest time resulted in Italy (0.4 months), as expected because the sponsor was Italian with an already good knowledge of national rules, and the longest time was in Greece (21.7 months), given that it was required to translate the protocol in the national language.

Overall, the parallel submission was efficacious in the EU, with the exception of Italy where the CA authorisation was granted after the EC approval, causing consistent delays for trial initiation. In Italy, at the time of DEEP-2 submission and until very recently, while the single opinion was recognised by the national law D.lgs. 211/2003 [15] implementing Directive 2001/20/EC [3], de facto the ‘satellite ECs’, in charge of the acceptance or refusal of the single opinion granted by the “coordinator EC”, were used to review the CTA documents in line with their own internal procedures. With regards to the CA, the legal officer of the trial site was addressed after the ethics approval. In fact, while the above mentioned directive [3] as well as the Italian law itself dealt with the “tacit authorisation”—*if the Competent Authority has not informed the sponsor of any objections within 60 days (with the mentioned exceptions)*—in practice in Italy a trial may not start if the legal officer has not provided the written authorisation! In fact, for many Italian satellite ECs the time necessary to complete the authorisation process was longer.

It should be underlined that, even if in the non-EU countries’ GCP provisions are not officially implemented in a national law, the type and number of documents required for the EC approval are similar to what is required in Europe. This represents the most interesting aspect of the DEEP experience documenting that it is possible to perform a GCP-compliant paediatric trial also in a heterogeneous framework, following the EU regulatory guidance as reference.

Moreover, provided that all the legal-specific requirements were met, the timing to obtain the final trial authorisation was significantly reduced in non-EU countries in comparison with the EU.

Regarding the EU, DEEP experience has also documented the need for simplifying and further harmonising the authorisation process.

In the EU, the provision for CA authorisation and EC approval timing is indicated in Directive 2001/20/EC [3] as 60 days for the single opinion release and 30 days for its acceptance. Overall, our analysis revealed that in the EU countries, the timeframes were not compliant with this requirement. Furthermore, these timeframes resulted in longer times than in non-EU countries.

The issues that affected the whole duration of the CTA in such a multi-national trial, resulted in the long lists of documents for submission, the requirement applicable in Italy and the UK to use separate portals from EudraCT to carry out the CTA and prepare the CTA form, and the queries raised from CAs and ECs.

Differences exist regarding the documents for submission, notwithstanding the EU rules [3] and guidance [5, 6].

Important issues for children’s protection were found in Italy: relevance of the trial and trial design, benefit/risk evaluation, foreseen risks, justification for including minors (if not given in the protocol) were required by law [15]. A recent Italian law [15] has established that insurance for clinical trials in children must foresee at least 10 years from completion of the clinical trial as the minimum period of tail coverage for the risk; this is the minimum time required to ascertain their regular psychophysical development.

We demonstrated that queries impacted on the timing for getting the ECs opinion and the CAs authorisation in the EU. For example, the longest time for obtaining the full authorisation in Cyprus (32.9 months) was due to requests related with the protocol from the EC. In contrast, no additional queries were posed in non-EU countries where the approval process was quick and easy.

After the CTA of DEEP-2, some changes in the national regulatory frameworks occurred. For example, in Italy. Laws n. 189/2012 (“Balduzzi Decree”) [20] and 158/2012 [21] established the reduction of national ECs, and the Italian Medicines Agency (AIFA) became the national CA. Outside the EU, in Albania the first law specifically dealing with clinical trials and partially aligned with the EU Directive [3] was released in 2014 [22].

Well-known differences exist to collect the informed consent at the national level [1]. Even if all countries acknowledged the need for collecting informed consent from parents or legal representatives, in Albania, Cyprus, Greece, Italy and Tunisia, both parents must consent for the participation of a child in the trial (contextually or in different moments), while in the UK and Egypt the signature from only one parent is accepted. This issue would be impracticable to harmonise, even in the light of a common EU regulation.

Part of the queries raised by ECs dealt with the informed consent/assent process. Our data show that the assent form was mentioned in the list of documents to be provided for the CTA in Italy, UK and Egypt, but in no country was the assent form and information material for paediatric subjects to being mandatory by law. Of note, Egypt was the only non-EU country to list the assent among the documents to be submitted.

On this specific topic, notwithstanding that DEEP provided common ad hoc material for the informed assent (three different age-tailored informative booklets and two assent forms prepared with the contribution of the patient representatives, shared among partners and made available in six languages [14], based on the EC Recommendations [8], the position of the involved countries was demonstrated to be different: in the UK, the age-tailored booklets prepared by the sponsor with the support of a communication team (one for pre-school children with coloured figures, one for school-age children with figures and friendly childish explanations, and one for adolescents with more complete information and images adjusted for this age range) were not accepted by the EC as considered too complicated for school children and not suitable for adolescents.

For other issues, very different points of view were expressed, and therefore it was necessary to adopt ad hoc solutions to respect national social and religious habits. This was the case of contraception. Two ECs in Italy required more details on this issue both in the documents for both girls and boys. The EC in Greece required insurance coverage for contraception failures. In the UK, the information sheets for adolescents had to reflect the course of action if a young person was pregnant. In Egypt, the ECs asked to remove all details on contraception in the consent and assent documentation.

All these issues represented a great challenge and resulted in a global delay in starting the authorisation process. These issues were solved through a strong interaction among investigators, DEEP coordination staff and ECs according to the DEEP project procedures.

In the UK, the two booklets for older patients (6–10 years and 11–17 years) were replaced by a single information sheets. In Egypt, the term”contraception” was avoided in the information material for patients but considered in the oral consent procedure to parents.

So, what to do to improve this situation?

Ad hoc actions to support the acceptance of the Ethical Recommendations [9] provisions should be done, including educational and patient empowerment initiatives. These could facilitate the presence of “expert patients” being able to contribute to the preparation of information material for informed consent and access process and favour a constructive dialogue with the ECs.

Improvement is expected from the already issued but not yet applicable CT Regulation [13], whose major aim is to overcome the drawbacks surrounding clinical research and the harmonisation among countries. However, this Regulation is not designed to affect the national ethics approval. Therefore, we will still need to know and address local requirements.

Some help could derive from the involvement of large research consortia able to deal with the differences and to set up a strong collaboration not limited to the scientific topics but including also cultural and social issues and the direct contribution of patients.

Accordingly, networks on paediatric research acting at the international or global level will be crucial. The first initiative to establish a global rare disease CTs network is under discussion at the FDA level as a “Rare Disease Cures Accelerator” [23]. By providing a more centralised infrastructure and common platform(s), this tool would support the conduct of CTs in rare disease populations.

All things considered and in spite of the wide regulatory, cultural and linguistic heterogeneity, this has represented a useful case study to highlight the existing differences and difficulties and also demonstrates that a GCP paediatric study is possible even in a heterogeneous context.

### Strengths and limitations

Strengths:

- This work represents the first available report on the clinical trial application in a multi-national framework including a descriptive approach and statistical analysis;
- This study provides a real life-screenshot of the EU rules that may be useful for the upcoming application and implementation of the new Clinical Trial Regulation (CTR).

Limitations:

- The sources considered for national/local provisions and procedures to authorise a paediatric trial in the considered countries as detailed below in the methods section might not be exhaustive due to local requirements available in the national languages.

## Conclusions

The effort to submit a paediatric multi-centre multi-national clinical trial in the rare disease setting is not easy.

In line with previous data [1], unfortunately the procedures and requirements to carry out a CTA are not harmonised across Europe and this impacts on the time for starting the trial.

DEEP-2 study represents a unique GCP-compliant active-controlled multi-national paediatric trial involving EU and non-EU countries [24] in which ad hoc measures were in place to overcome the regulatory and ethical differences among countries and sites, often delaying or preventing the starting of such a trial.

Based on our practical experience, we recommend the following actions for a multi-national CTA:

- Setup of a single submission package adaptable to different local situations,
- Deep knowledge of local requirements,
- Strong interaction among investigators, sponsor and ECs, not limited to the scientific topics but including also cultural and social issues,
- Direct contribution of patients,
- Involvement of large research consortia able to deal with the differences, and.
- Engagement of international networks on paediatric research.

In the meantime, we will wait for the real outcomes of the new Regulation to solve the gaps and challenges faced in such a multi-national clinical trial. In the paediatric setting, this will be harder as further rules should be complied with.

## Supplementary Information

**Additional file 1**. Supplementary material.

## Data Availability

The datasets used and/or analysed during the current study are available from the authors on reasonable request.
