# Supplementary material for: Ethical and procedural issues for applying researcher-driven multi-national paediatric clinical trials in and outside the European Union: the challenging experience of the DEEP project
Source: BMC Med Ethics. 2021 Apr 29;22:49. doi: 10.1186/s12910-021-00618-2 (PMC8086274; doi:10.1186/s12910-021-00618-2)
Supplement: Supplementary file 1 — Additional file 1. Supplementary material. [file 12910_2021_618_MOESM1_ESM.docx]

1. **Table 1 – Questionnaire on necessary national information for clinical trials authorization used in the context of the survey addressed to DEEP-2 local study teams**

| **Laws governing clinical trial application** |  |
| --- | --- |
| **Requirements for minors information** |  |
| **Provisions for data protection and confidentiality** |  |
| **Name of all the competent Ethics Committees to be involved in the submission of DEEP trial** |  |
| **Name of all the Competent Authorities to be involved in the submission of DEEP trial** |  |
| **Nationally requested documents to be submitted** |  |
| **SOURCES** |  |

1. **Table 2 - National/local CTA procedures**

| **Country** | | **CTA form preparation** | **N. sites** | **CA/EC submission** | **N. CAs addressed** | **N. ECs addressed** |
| --- | --- | --- | --- | --- | --- | --- |
| **EU** | **Cyprus** | Through EudraCT | 1 | parallel | 1 | 1 |
|  | **Greece** | Through EudraCT | 1 | parallel | 1 | 1 |
|  | **Italy** | National database (OsSC) | 12 | CA after EC | 12 | 12 |
|  | **UK** | National database (IRAS) | 3 | parallel | 1 | 1 |
| **Non-EU** | **Albania** | n.a. | 2 | parallel | 1 | 2 |
|  | **Egypt** | n.a. | 3 | CA after EC | 1 | 3 |
|  | **Tunisia** | n.a. | 1 | CA after EC | 1 | 1 |

1. **Table 3 - General Linear Model (GLM) analysis: Ethics Committees approval time versus**
2. **EU or non-EU countries, number of requests raised by Ethics Committees**
3. **and Ethics Committee site (Coordinator vs satellite).**
4. The table shows the effects of the number of EC requests on the means of ethics approval time stratified by EU or non-EU countries and coordinator/satellite EC.

| Tests of Between-Subjects Effects | | | | | | |
| --- | --- | --- | --- | --- | --- | --- |
| Dependent Variable: ECs approval time (months) | | | | | | |
| Source | Type III Sum of Squares | df | Mean Square | F | Sig. | Partial Eta Squared |
| Corrected Model | 257.755 | 3 | 85.918 | 8.461 | .001 | .613 |
| Intercept | 43.583 | 1 | 43.583 | 4.292 | .055 | .212 |
| Number of ECs requests | 200.209 | 1 | 200.209 | 19.716 | .000 | .552 |
| EU/non-EU countries | 7.825 | 1 | 7.825 | .771 | .393 | .046 |
| Coordinator/satellite EC | 10.679 | 1 | 10.679 | 1.052 | .320 | .062 |
| Error | 162.474 | 16 | 10.155 | - | - | - |
| Total | 881.990 | 20 | - | - | - | - |
| Corrected Total | 420.230 | 19 | - | - | - | - |
| R^2^ = .613 (Adjusted R^2^ = .541). | | | | | |  |

******

**Figure 1 - Cluster Analysis: distance from cluster centre.** The figure shows the Euclidean distance between each site and its classification centre. The numbers behind the country name are the site numbers. The classification centre, or centroid, of the cluster is the pair (EC time approval, CA time authorisation) containing the means of the two variables in that cluster. It reveals that all the sites belonging to the first group are in EU countries, and the ones belonging to the second are in non-EU countries. In particular, among the 20 cases (single CTA site), 15 were assigned to the first cluster (EU countries) and 5 to the second cluster (non-EU countries).. Longer authorisation period for the cases in the first cluster (EU) and shorter authorisation period for the cases in the second cluster (non-EU).
